# Cancer 3D Models for Metallodrug Preclinical Testing

**DOI:** 10.3390/ijms241511915

**Published:** 2023-07-25

**Authors:** Diogo M. Engrácia, Catarina I. G. Pinto, Filipa Mendes

**Affiliations:** 1 Center for Nuclear Sciences and Technologies, Instituto Superior Técnico, Universidade de Lisboa, 2695-066 Bobadela LRS, Portugal; diogo.engracia@tecnico.ulisboa.pt (D.M.E.); catarina.pinto@tecnico.ulisboa.pt (C.I.G.P.); 2 Department of Nuclear Sciences and Engineering, Instituto Superior Técnico, Universidade de Lisboa, 2695-066 Bobadela LRS, Portugal

**Keywords:** cancer, 3D models, metallodrugs, precision medicine

## Abstract

Despite being standard tools in research, the application of cellular and animal models in drug development is hindered by several limitations, such as limited translational significance, animal ethics, and inter-species physiological differences. In this regard, 3D cellular models can be presented as a step forward in biomedical research, allowing for mimicking tissue complexity more accurately than traditional 2D models, while also contributing to reducing the use of animal models. In cancer research, 3D models have the potential to replicate the tumor microenvironment, which is a key modulator of cancer cell behavior and drug response. These features make cancer 3D models prime tools for the preclinical study of anti-tumoral drugs, especially considering that there is still a need to develop effective anti-cancer drugs with high selectivity, minimal toxicity, and reduced side effects. Metallodrugs, especially transition-metal-based complexes, have been extensively studied for their therapeutic potential in cancer therapy due to their distinctive properties; however, despite the benefits of 3D models, their application in metallodrug testing is currently limited. Thus, this article reviews some of the most common types of 3D models in cancer research, as well as the application of 3D models in metallodrug preclinical studies.

## 1. Introduction

Animals as models of human anatomy, physiology, and disease pathology have been a key tool for scholars for millennia, allowing for an understanding of the human body and disease without risking human lives [1]. Even though animal models are fundamental tools in modern biomedical research for proving scientific significance, their use is being reduced and, when possible, replaced as a result of animal ethics. An ethical framework has been implemented to reduce animal usage as well as the suffering caused while doing research. Additionally, projects involving animal models must be evaluated in order to determine if the costs outweigh the benefits [2]. Besides the ethical concerns, scientific issues also need to be considered, particularly in the biological evaluation of novel drugs, as the genetic differences between animal models, namely mice, and humans, may lead to results without strongly supported translational significance [3,4,5].

Therefore, in vitro models such as two-dimensional (2D) cell cultures, arose in the 20th century as possible alternatives for the biological studies of normal gene function, disease states, therapeutic strategies, etc. [6]. Due to their simplicity and affordability, 2D monolayer cultures are considered extremely appealing for laboratory use [7]. However, these cultures lack the three-dimensionality of tissues and organs, leading to alternate tissue architecture, cell differentiation, cellular communication, and biochemical signaling in comparison with what is found in vivo [5,8]. Nonetheless, these models are still useful for a variety of cell growth and functional studies and for the production of biological therapeutics [7,9].

Three-dimensional (3D) models can be presented as a step further in biomedical research, allowing for a higher throughput and mimicking of human characteristics than animal models, while maintaining more accurate tissue signaling than 2D models [4,5]. These cell models are capable of better simulating tissue complexity, namely cell morphology, cell differentiation, gene expression, interactions between the cells and the extracellular matrix (ECM), concentration gradients, tissue stiffness, and drug response [10,11,12]. Furthermore, 3D culture models may also allow for a reduction in animal models used while still recapitulating the in vivo microenvironment, which is missing in more simple in vitro models [13]. These more advanced culture systems can be applied to several areas of fundamental and translational research, for instance, stem cell biology, toxicology, and developmental biology. 

## 2. 3D Models in Cancer Research

Cancer occurs when cells accumulate genetic errors, proliferating out of control and possibly spreading to other areas of the body, forming metastasis [14]. Regarding data from 2020, it is estimated that there were around 19.3 million new cases of cancer worldwide and that nearly 10 million people died from cancer, making it the second leading cause of death around the globe [15]. Besides the major impact that cancer has on people’s lives, it also represents a notorious economic impact on the whole of society. In 2020, it is estimated that the United States of America spent around 194 billion Euros on cancer care [16]. Additionally, in Europe (27 countries of the European Union, Iceland, Norway, Switzerland, and the United Kingdom), the overall cost of cancer was 199 billion Euros in 2018 [17]. Cancer prevention, diagnosis, and treatment are one of the 21st century’s most important public health concerns, as demonstrated, for instance, by the EU’s “Europe’s Beating Cancer Plan”, launched in 2021 [18], and the USA’s “Cancer Moonshot”, launched in 2016 [19], which aim to address, among others, a better understanding of cancer biology and the development of better therapies and diagnostic methods. 

Cancer cells exist in a complex cellular environment, the tumor microenvironment, which is a key modulator of cancer cell behavior [20,21]. Tumors are not only composed of cancer cells, which possess several genetic mutations that affect proper cellular functioning, but also of other cells that are present or recruited to the tumor’s microenvironment, such as fibroblasts, immune cells, and endothelial cells. Moreover, the tumor’s microenvironment is also composed of non-cellular components, including growth factors, ECM, cytokines, and chemokines, which act as modulators of cancer development [22,23]. The molecular communication established between the aberrant and normal cells, as well as the interactions with the ECM, modulates the overall tumorigenicity [24,25,26]. Because of these interactions, several molecules that affect crucial cellular events, such as proliferation, angiogenesis, migration, and tissue remodeling, among others, are released, creating an environment that favors cancer progression [27]. Furthermore, transmembrane cell adhesion molecules sense and transmit information regarding ECM alterations, activating signaling pathways and regulating cancer cell behavior [28].

The complexity of the tumor’s microenvironment and the interactions established with cancer cells are the main reasons 2D models fail to accurately replicate the in vivo tissue [29]. Even though 2D monolayer cell cultures have allowed for breakthrough discoveries in fundamental cancer biology, these are grown in simple and unrealistic conditions, where the cells are attached to rigid surfaces and lack three-dimensionality [30,31]. The cells grown in a 2D culture become deformed in the out-of-plane axis and are not mechanically or physically restrained, which may lead to cell behavior changes [32]. The application of these models in anti-cancer drug development hinders the efficiency of the process, leading to erroneous results [33]. This happens because drug optimization is based on conditions not found in vivo, considering that 2D models are unable to accurately mimic the microenvironment of the tumor [34,35]. Using these simple models for drug screening results in numerous drugs that do not reach the market and, consequently, pharmaceutical companies’ investment losses [36]. 

Considering the 3D models’ capacity to more accurately replicate the tumor microenvironment, the interest in applying these for drug development has increased over the year [37,38]. Moreover, the introduction of 3D models in drug development may also allow for a reduction in animal models, addressing some ethical concerns [13]. However, these should not be seen as the only correct tool for cancer research, but as a support approach to be used alongside 2D models before in vivo testing in animal models [39,40].

## 3. Types of 3D Models in Cancer Research

**Figure 1:**
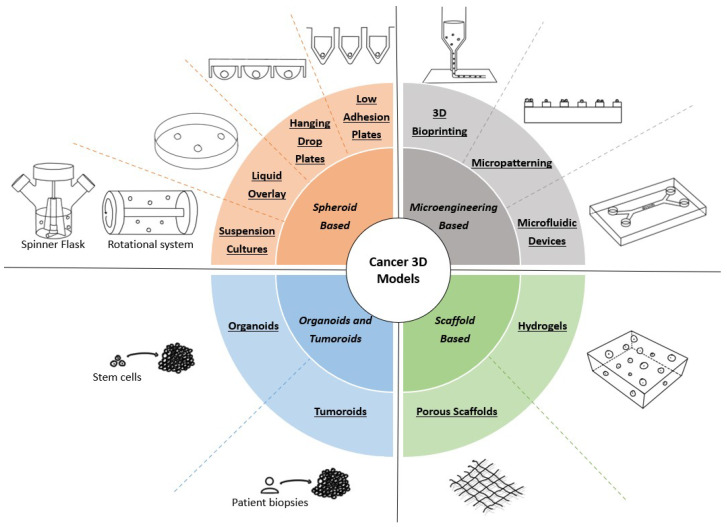
Types of 3D models used in cancer research. Spheroid-based models promote the formation of spheroids through the self-assembly of tumor cells using several methodologies, such as medium agitation or culture in nonadherent conditions. Microengineering-based models implement micro- and nanotechnologies to obtain systems that allow for precisely controlling important variables. Scaffold-based models rely on biomaterials to produce a structure that mimics the in vivo ECM, which is used to support cancer cell growth and proliferation. Organoids and tumoroids are in vitro self-assembling aggregates derived from stem cells and patient biopsies, respectively, allowing for obtaining models with more than one cell type.

When applying a 3D model to cancer research, one must consider that each system presents its advantages and limitations, as none are capable of mimicking integrally the in vivo tissue [5,11]. Overall, the 3D models may be divided into several major categories: spheroid-based, microengineering-based, and scaffold-based categories, and organoids and tumoroids. A graphical summary of each 3D model can be found in Figure 1, while Table 1 presents the main advantages, disadvantages, and complexity (evaluated from low to high) of each cancer 3D model. Besides these types of cellular 3D models, explant-based 3D models have also been applied to cancer research. Precision-cut tissue slices (PTCS) are a type of 3D tissue explant derived from human or animal organs. Notwithstanding their ability to closely mimic in vivo conditions, explant-based models such as PTCS still present ethical questions regarding the use of animal models [41,42]. Thus, we will not explore the details of this type of cell model more deeply in the present review.

### 3.1. Spheroid-Based Models

Multicellular tumor spheroids (MCTS) are 3D models that resemble micrometastases or microregions of a tumor, comprising peripheral proliferating cells, deep non-proliferating cells, and, in some cases, a necrotic core, obtained through the self-assembly of tumoral cells [43,44,45]. Additionally, the geometry of spheroids allows for studying different subpopulations of cancer cells in relation to their microenvironment [45]. In comparison with 2D monolayer models, spheroids are able to better recapitulate in vivo aspects, such as microenvironment interactions, tissue architecture, gene expression, hypoxic regions, and metabolism [46,47,48,49]. As a result of their similarities with in vivo tumors, MCTS have been extensively used in research, in particular for the evaluation of different therapeutic strategies [47,50,51]. 

Despite their several advantages, spheroids also present some limitations, such as imaging difficulties using fluorescence microscopy due to the spheroid thickness and light-scattering phenomenon. This makes high-resolution images of intact spheroids, particularly from their interior, difficult to obtain [52,53]. This could be addressed by cutting the spheroids into thin slices; however, it may damage the spheroid structure and result in the introduction of artifacts during computational analysis [12,52]. Alternatively, optical sectioning microscopes, such as confocal laser scanning microscopy (CLSM), multiphoton microscopy (MPM), and light-sheet fluorescence microscopy (LSFM), can be used to image the deeper regions of intact spheroids [54,55].

Several methods for the efficient growth and manipulation of spheroids derived from rodent and human cancers have been established [56,57,58]. The four principal approaches for spheroid production are suspension cultures, hanging drop plates, liquid overlay technique, and low adhesion plates.

#### 3.1.1. Suspension Cultures

In suspension-based or agitation-based approaches, a cell suspension is maintained in motion through stirring or rotation. As the suspended cells are constantly moving, they do not attach to the container walls, but instead develop cell−cell interactions, forming spheroids [59]. This type of culture can be performed in spinner flask bioreactors or rotational culture systems [60,61]. 

Spinner flasks are a simple way of producing spheroids in higher quantities, depending on the size of the bioreactor [62]. Moreover, spinner flasks allow for the control of culture properties, such as culture duration, while constant agitation promotes the transport of nutrients and waste products [59,63]. However, using spinner flasks involves a constant cell motion, resulting in a shear force that may have a negative impact on cellular physiology, as well as a larger volume of culture medium, making this technique more expensive than other methods [64,65]. Rotational systems present several similarities with spinner flasks, both in terms of their advantages and disadvantages [59]. However, contrary to the spinner flask, rotational systems present the advantage of having low shear forces, as in this method, both the container and its content are rotating [61]. These two techniques have advantages such as easy maneuvering, affordability, and the ability to scale-up production. However, heterogeneous spheroids regarding size and morphology are limitations that need to be considered, as the manual selection of spheroids may be necessary for drug screening [59].

#### 3.1.2. Hanging Drop

Hanging drop plates benefit from the fact that when no surface is available for cell adhesion, cells self-aggregate into spheroids. This is possible using a traditional tissue culture petri dish, where the lid is used to place small droplets, in which the spheroids form [66]. Alternatively, specialized plates with bottomless, open wells that allow for the formation of a small media droplet, can also be used [11]. Kelm et al. described a protocol using hanging drop plates that allows MCTS growth from a variety of cell lines, including some that are not frequently obtained with other methods. The spheroids produced present reproducibility, rapid generation, and homogenous size distribution, qualities that are essential for drug screening assays [58]. This last feature in particular is crucial, ensuring that the spheroids can be used immediately without the need to select the appropriate spheroids [67]. Furthermore, with these types of plates, it is possible to use the spheroids in an automated drug-screening platform, which may be relevant to the pharmaceutical industry [58]. However, due to plate incompatibility with microscopes and uncentered spheroids in the well plates, hanging drop plates can be difficult to image [68]. Moreover, media exchange is a difficult process in traditional plates, whereas specialized open-top plates are costly and difficult to adapt to the experimental requirements [59].

**Table 1:** Cancer 3D models in metallodrug preclinical testing: a summary of the advantages, disadvantages, and complexity (from low to high) of each 3D model, and examples from the literature of the application of these 3D models in this context.

Model	Advantages	Disadvantages	Complexity	Examples
***Suspension*** ***Cultures***	Transport of nutrients and waste; Easy maneuvering, affordability, and the ability to scale-up	Shear forces (spinner flasks); Larger volume of culture medium; Heterogeneous spheroids	Low	--
***Hanging Drop Plates***	Reproducibility, rapid generation, and homogenous size distribution	Difficulties imaging; Media exchange (traditional plates); Expensive (specialized plates)	Medium-low	--
***Liquid *** ***Overlay***	Quickly produce single, compact spheroids with a uniform size on a large scale; Easiness to manipulate the spheroids	Requires coating of the plate	Low	[69]
***Low *** ***Adhesion Plates***	Quickly produce single, compact spheroids with a uniform size on a large scale; Easiness to manipulate the spheroids; Pre-coated plates	Cost	Low	[70,71,72,73,74,75,76]
***Porous *** ***Scaffolds***	Enhanced nutrient delivery via interconnected pores	Thickness and non-transparency	Medium	--
***Hydrogels***	Similar mechanics, chemistry, and structural properties to in vivo tissues	Weak mechanical properties; Batch-to-batch variation; Rapid degradation	Medium	--
***3D*** ***Bioprinting***	Usage of bioinks with components of the tumor microenvironment; Vascularization; Modular and standard construction	Unwanted biological interactions, bioink-printing compatibility, resolution, and low efficiency	Medium-high	--
***Micropatterning***	Recapitulate the structure of the microenvironment; Control cell shape and overall tissue architecture	Generating stencils, with small feature sizes using soft lithography	Medium	--
***Microfluidic*** ***Devices***	Controlled release of several molecules; Affordability and transparency	Specific equipment; Low number of cells for assays	Medium	--
***Organoids***	Recapitulate the structure and different cell types of the tissue/organ	Exogenous ECM; Trained staff	High	--
***Tumoroids***	Recapitulate the patients’ tumors more accurately; Precision medicine potential	Exogenous ECM and external growth factors; Highly trained staff	High	[77,78]

#### 3.1.3. Liquid Overlay Technique and Low Adhesion Plates

In the liquid overlay technique, cells are cultured on a nonadherent surface and centrifuged to induce spheroid formation [48,79]. Nevertheless, it is necessary to take into consideration that this technique differs according to the type of plate used [57,80,81] and that the plate surface has to be coated with nonadherent materials, such as agarose or poly-HEMA, to induce spheroid formation [57,82]. This protocol allows for quickly producing single, compact spheroids with a uniform size on a large scale that are suitable for high-throughput assays. Another significant feature of this method is the easiness to manipulate the spheroids for further cultivation or analysis. MCTS from more than 20 different tumor cell lines were already established using this method. Nonetheless, there are certain cell lines, such as MDA-MB-231, MDA-MB-468, SK-BR-3, and MDA-MB-361, which do not form round spheroids using this technique, a problem that can be solved using a reconstituted basement membrane extract [57]. A commercial alternative of this basement membrane extract is Matrigel^®^, a basement membrane preparation derived from Engelbreth−Holm−Swarm (EHS) mouse sarcoma, rich in laminin, collagen IV, entactin, heparan sulfate proteoglycan, and growth factors, in order to induce the formation of an organized 3D structure. Similar to the liquid overlay method, low adhesion plates are able to promote spheroid formation due to the absence of cell attachment surfaces [11]. These pre-coated low-adhesion plates have the advantage that, in most cases, prolonged cultures and experimental assays can be performed without the need to transfer the spheroids to another type of plate [11]. In comparison with the liquid overlay technique, low-adhesion plates are easier to manipulate, as the plates are already pre-coated, although this aspect results in a higher cost [59]. Regardless of cost, low adhesion plates are the superior method for drug screening of all spheroid manufacturing methods.

### 3.2. Microengineering-Based Models

The implementation of micro- and nanoscale technologies with biomaterials has created great prospects for the development of 3D models for cancer research. Through this combination, it is possible to precisely control important variables involved in tumor growth, such as cellular spatial organization, biochemical gradients, and mechanical properties, among others [83]. Techniques such as micropatterning, 3D bioprinting, and microfluidics have been used as platforms for the discovery and preclinical evaluation of anti-tumoral drugs due to their exceptional adaptability in creating precise experiments [84].

#### 3.2.1. Micropatterning 3D Models

Controlling cellular adhesion, shape, and spread based on the control of the spatial features of the culture surface is the aim of micropatterning techniques [85]. The micropatterning of surfaces allows for recapitulating the structure of the microenvironment found in vivo, making it possible to further understand the correlations between microarchitecture and cellular function [86]. Using these approaches, it is possible to precisely position the cells in certain areas of the substrate, allowing for the control of its shape and the overall tissue architecture [87]. The most often used micropatterning procedures include seeding cells on a surface with regions of varied adhesiveness, which correspond to different patterns [88], and depending on the application and size of the cells to be patterned, these patterns can range from a few microns to hundreds of microns. Micropatterning models have been used to assess the efficacy of anti-tumoral drugs and to study cancer development at the microscale level [89,90,91]; however, generating stencils, which are used to generate the patterns, with small feature sizes using soft lithography, is a significant limitation of this technique [85].

#### 3.2.2. 3D Bioprinting Models

Three-dimensional bioprinting consists of building 3D structures by accurately arranging biological and biochemical elements layer by layer [7], and allows for the production of interesting and accurate tumor models using bioinks, which contain cells, cytokines, and biomaterials similar to the in vivo ECM [92]. There are several methods of 3D bioprinting, such as biomimicry, where one attempts to recreate the tissue found in vivo; autonomous self-assembly, which simulates organ development through the manipulation of tissue genesis and organogenesis; and mini-tissue building blocks, which consist of building smaller constructs and combining them into a larger structure. Additionally, material deposition can be performed through inkjet, in which a nozzle produces droplets that are precisely placed on a scaffold; extrusion, similar to inkjet but for viscous materials; and laser-assisted printing, where an energy-absorbing layer is exposed to a laser beam, causing a bubble to form in the bioink solution [93]. 

Contrary to scaffold-based 3D models, bioprinting technology provides a greater vascularization because of the process’s reliability, scalability, and enhanced control over vascular growth, resulting in the design of scaffolds that better represent tumor microenvironment heterogeneity and in the creation of superior 3D in vitro models of cancer [7,94]. Additionally, 3D bioprinting technology enables the modular and standard construction of in vitro models for a high-throughput method for personalized drug screening [95]. Some limitations inherent to 3D bioprinting are related to the materials used and the manufacturing process, such as unwanted biological interactions, bioink-printing compatibility, resolution, and low efficiency [96]. Another aspect that has to be further studied is the development of bioinks that are biocompatible, non-toxic, do not induce immune responses, and properly support the cells for each application [93]. Nevertheless, several platforms based on 3D bioprinting have been used to test anti-tumoral drugs and for cancer basic research [97,98].

#### 3.2.3. Microfluidic Models

In microfluidic devices, cells are cultured in chambers with dimensions on the micrometer scale [99]. These devices are probably mostly used for perfusion-based cell cultures, allowing for a consistent supply of oxygen and nutrients while also eliminating waste, providing an environment similar to the one found in vivo [11]. Furthermore, in perfusion systems, the culture medium perfusion recapitulates the shear forces caused by blood flow in vivo [11]. However, besides the perfusion-based microfluidic devices, static models have also been developed for cancer research [100,101]. 

These microfluidic systems allow for the culture of multiple cell types, which can be separated by porous membranes or cultured in different chambers, in order to obtain a more complex tumor model where cell interactions can be recapitulated [102,103]. Additionally, microfluidic devices can be adapted to study several biological changes that occur in cancer, such as metastasis and angiogenesis [104]. A major advantage of microfluidic devices is the capacity to control the tumor microenvironment, allowing for a controlled release of several molecules, such as growth factors and nutrients [7,105]. Microfluidic systems have other advantages, such as affordability, transparency, and the power to establish a physiologically accurate environment, making them suitable alternatives for anti-tumoral drug testing [84]. Overall, microfluidic devices are able to mimic different aspects of the tumor microenvironment, such as vasculature, pressure, co-culture, shear stress, and oxygen and nutrient gradients [106]. Despite being an interesting platform for high-throughput drug screening, the manipulation of microfluidic devices requires specific equipment and the number of cells cultured may be lower than what is needed for certain assays [107]. To overcome this last problem, several laboratorial assays have been adapted to be compatible with lower cell numbers, and systems that are able to produce higher cell numbers have also been developed [7,108].

### 3.3. Scaffold-Based Models

Scaffolds are 3D biomaterials designed to resemble the ECM, promoting cellular interactions and proliferation while remaining non-toxic. Scaffolds can be used to produce spheroid-like aggregates, or their surface can be functionalized in order to allow for cell adhesion [36]. The 3D scaffold-based models are particularly used for cancer cell proliferation and to mimic the complexity of the tumor microenvironment, being an interesting tool to study cellular functions and interactions [109,110]. These biomaterials can be produced from natural or synthetic materials, such as chitosan and Poly(lactic-coglycolic acid) (PLGA), respectively, according to the desired application. Additionally, hybrid materials, made of biological and synthetic components, can also be used, such as poly(ethylene glycol) (PEG) associated with collagen and Matrigel^®^ [111,112]. Porous scaffolds and hydrogels are two scaffolding techniques commonly used [113].

#### 3.3.1. Porous Scaffolds

Porous scaffolds can have a sponge, foam, or mesh appearance, and their porosity enables cell seeding and adequate nutrient exchange [113,114]. Porous scaffolds provide a surface for cells to adhere to and build their own ECM, as well as enhance nutrient delivery via interconnected pores, lowering the likelihood of a necrotic center forming [114]. However, this type of scaffold presents thickness and non-transparency as its key disadvantages, making observing biological events through high-content imaging approaches quite challenging [36]. 

#### 3.3.2. Hydrogels

Hydrogels are cross-linked three-dimensional networks of hydrophilic polymers that are rich in water content and are important for the development of 3D cancer models due to their ability to mimic the in vivo ECM [115]. In this type of scaffold, cells can be grown on top of the matrix after it has solidified, or they can be mixed with the liquid hydrogel and embedded inside the matrix during gelation [7]. Despite being interesting for their similar mechanics, chemistry, and structural properties to in vivo tissues, hydrogels still have some limitations [7]. Similar to other scaffolds, hydrogels derived from natural materials, such as collagen or Matrigel^®^, have weak mechanical properties, may be subject to batch-to-batch variation and rapid degradation, and can result in immunogenic responses [116]. Therefore, synthetic materials, such as PEG, present themselves, in some cases, as a better alternative, allowing for better reproducibility, control over microenvironmental cues, and molecule transport [115,117,118]. Nevertheless, synthetic hydrogels may present some problems, such as the availability of oxygen, discrepancy in the distribution of soluble growth factors, microenvironment variability, and imaging and cell analysis complications [116]. Another important issue is that scaffold-based 3D models have a low propensity for vascularization, which is crucial in cancer development research [92].

### 3.4. Organoids and Tumoroids

Organoids are in vitro aggregates derived from stem cells that are capable of self-organization, forming a structure similar to an organ with several cell types while maintaining similarities with the in vivo tissue. The utilization of organoids in research aims to replicate the organ’s structure, organization, and function [119,120]. Despite several protocols that have been established for the production of cancer organoids, as far as we know, all of them require exogenous ECM [36,121,122]. This represents a limitation as it is known that cancer development triggers several biological changes, such as ECM remodeling, which affects drug resistance [36].

Tumoroids are organoids derived from patient biopsies that are able to recapitulate the patients’ tumors more accurately as they preserve the genetic characteristics and heterogeneity of the original tumor, hence better capturing inter-patient heterogeneity [36,123]. This makes tumoroids a crucial model for studying rare cancers for which there are no immortalized cell lines [36,124]. Tumoroid growth protocols for several types of cancer have been established [78,125,126]. Similar to some organoid protocols, tumoroids need to be supplemented with external growth factors and be in the presence of exogenous ECM, such as Matrigel^®^ [36,127]. An important advantage of using tumoroids is that drug response in these models more accurately represents the patient’s drug response and thus may allow for a personalized therapy selection [128]. A drawback of this model, as well as organoids, is the need for highly trained staff to perform patient cell isolation and manipulation [36]. Moreover, these cell models may be prone to ethical concerns, regarding the source of the stem cells, and the informed consent and privacy of cell donors. Nevertheless, when comparing these models’ advantages and disadvantages, the potential outcomes are considered to outweigh the ethical questions when these are properly addressed [129].

As mentioned before, cancer 3D models can be applied to various fields of research and development, such as the preclinical testing of metallodrugs. Next, we describe and discuss the importance of metallodrugs in cancer, as well as the application of cancer 3D models for the preclinical evaluation of these types of drugs.

## 4. Metallodrugs in Cancer

Currently, there are several targeted therapies available to treat different types of cancer, for example, anti-hormonal treatments and monoclonal antibodies against cancer cell-specific receptors. Nevertheless, conventional chemotherapy and radiotherapy remain the key tools for cancer treatment. Thus, there is an unmet need to develop innovative and improved pharmacological options for cancer therapy [130,131], but the design and development of effective anti-cancer drugs with high selectivity, minimal toxicity, and reduced side effects is still a challenging endeavor [132,133]. 

Metal-based compounds have been used throughout history for their therapeutic properties [134]. Despite the widespread use of this type of compound, the absence of a clear demarcation between therapeutic and hazardous dosages posed a significant obstacle [135]. In the 1960s, the “accidental” discovery by Barnett Rosenberg of cisplatin (cis-[Pt(NH_3_)_2_Cl_2_]), a platinum (Pt) compound, paved the way for modern metal-based anti-cancer drugs [136,137]. Besides platinum, research regarding the application of other transition metals, such as ruthenium (Ru), gold (Au), silver (Ag), iron (Fe), copper (Cu), arsenic (As), and cobalt (Co), as anti-tumoral compounds have gained increasing interest among the scientific community, and a wide range of metal-based compounds has been developed [138,139,140,141,142,143,144]. 

Transition metal complexes have been particularly explored for therapeutic applications in cancer due to their distinctive properties such as redox activity, various coordination modes (allowing for the design of different metal complexes), d orbitals partially filled (allowing for electrons to be easily removed or added to these orbitals, making them suitable to act as catalysts or to develop compounds with multiple oxidation states), and reactivity towards organic substrates (allowing for the design of drugs that preferentially interact with a biomolecular target) [145]. Additionally, metals have the important characteristic of forming positively charged ions in an aqueous solution, which can bind to negatively charged biomolecules. Therefore, the compound´s charge can be modified according to the coordination environment, directing the drug to the desired biological target [134,146,147,148]. Furthermore, the metal itself, the ligand, and the metal−ligand interactions endow different characteristics to metal complexes, and thus metal-based anticancer agents can be generally classified as functional compounds, structural compounds, metal ions as carriers of active ligands, metal compounds that behave as catalysts, and photoactive metal compounds. 

The chemical and anti-tumoral properties, as well as the potential mechanism of action of metallodrugs, have been extensively reviewed elsewhere, and the reader is referred to excellent publications for further information on this topic, which is out of the scope of the present review [149,150,151,152,153]. 

Despite the intense research in the design and biological evaluation of novel anti-cancer metallodrugs with very promising results that, in some instances led to advancement to clinical trials (particularly for Pt and Ru-based compounds), very few have been approved for clinical use. Cisplatin was approved in 1978 for the treatment of advanced bladder, testicular, and ovarian cancers [154], followed by two other Pt-based drugs: carboplatin, which started to be used in clinical practice in 1989 for the treatment of initial ovarian cancer, and oxaliplatin, which was approved in 1996, in Europe, and 2003, in the USA, for advanced colorectal cancer [144,155]. Other than these, the only metallic non-Pt drug that was clinically approved in the 2000s for cancer therapy is arsenic trioxide (As_2_O_3_) [144].

## 5. Three-Dimensional Models as Tools to Test Metallodrugs

When considering the preclinical testing scenario of different types of anticancer drugs such as organic and inorganic small molecules, antibodies, or biological-based drugs and metallodrugs, 3D cellular models present favorable characteristics that promote a more realistic evaluation of the antitumoral potential of these different classes of drugs, and potentially improve drug discovery and development. However, in the pharmaceutical industry, drug portfolios are increasingly diversified with only 40–50% being small molecules, and there are now a broad diversity of chemistries and mechanisms of action or toxicity to consider [156]. Therefore, for 3D models to become widely implemented as new preclinical models, there is still the need for a demonstration that their performance is reliable across a suitably broad set of compounds, such as large molecules and biologic therapies in a robust and repeatable manner, and the definition of ways for companies to implement the technology within routine preclinical workflows [156]. 

In the case of metallodrugs, despite the numerous advantages of 3D models described in the previous sections, the application of these platforms is still quite scarce. Conventional 2D and animal models appear to be the standard tools to test the biological response to metallodrugs. As mentioned previously, there is not an ideal model that is able to fully recapitulate in vivo tumor characteristics; therefore, 2D, 3D, and animal models should be incorporated into cancer research, each one allowing for evaluating different aspects of the therapeutic potential of a metallodrug. 

Particularly relevant for metallodrugs is the establishment of oxygen gradients within the 3D multicellular structure, as some complexes are redox active, and this feature allows for a more realistic evaluation of their predicted in vivo activity. Additionally, the availability of several analytical techniques suitable for the determination of the metal distribution and the metallodrug´s active species is an experimental advantage in the applicability of 3D models for this type of anticancer drug. Nevertheless, there are some important practical challenges in the adaptability of cancer 3D models for metallodrug testing that need to be acknowledged. For instance, suspension cultures might not be the ideal model for high-throughput assays, while some of the materials used in microengineering-based and coating-dependent models may absorb hydrophobic compounds, a common feature in several classes of metallodrugs. Several other important hurdles still need to be overcome, such as a lack of harmonization of protocols and assays, limited access to biological materials in uncommon tumor types, and particularly the time-sensitiveness o tumoroids due to the number of passages between tissue collection and drug testing.

To the best of our knowledge, most of the metallodrug evaluation studies that incorporate 3D models use spheroid-based models, as summarized in Table 1, which depicts the advantages, disadvantages, and complexity of each cancer 3D model, as well as published literature on the application of 3D models for metallodrug preclinical testing. This could be related to the fact that protocols for the spheroid production of several cancer cell lines have already been well established. In comparison with other 3D models, spheroids are relatively easy and inexpensive to obtain, while still providing a 3D structure that can replicate the aspects of in vivo tumors. More recently, some studies have already started incorporating organoid and tumoroid models in their research. The complexity of these models not only allows for better mimicking the in vivo tumor, increasing the relevancy of the study, but also introduces aspects of precision medicine, which are essential for future clinical applications. Additionally, microengineering approaches, especially 3D bioprinting, have gained interest lately, due to their ability to precisely control key aspects of experimental conditions. In the following sections, a brief description of the types of assays performed in these models will be presented, as well as the most illustrative examples of preclinical biological evaluation of metallodrugs in 3D models. 

### 5.1. Assays Performed on 3D Models

The application of 3D models to test the anti-tumoral activity of metallodrugs has mainly focused on the assessment of cytotoxicity, penetration, and evaluation of metabolic effects.

#### 5.1.1. Cytotoxicity Assays

Cytotoxicity can be defined as the toxicity induced by chemotherapeutic drugs in live cells [157]. The viability of 3D models can be assessed after incubation with the metallodrugs through several methods, such as the alamar blue assay, MTT (3-(4,5-dimethyl-2-thiazolyl)-2,5-diphenyl-2H-tetrazolium bromide) assay, CellTiter-Glo^®^ 3D Viability Assay, or acid phosphatase (APH) assay. These methods use different metabolic reactions to evaluate the number of viable cells in the 3D models after exposure to the metallodrug [70,71,72,73]. The alamar blue and MTT assays are based on the reduction of resazurin or tetrazolium salts, measuring the mitochondrial activity [158], while the CellTiter-Glo^®^ 3D Viability Assay indicates cell viability through ATP measurement [159] and the APH assay quantifies the cytosolic acid phosphatase activity [160].

#### 5.1.2. Penetration Assays

One of the factors responsible for drug resistance may be the inability of anti-cancer drugs to penetrate solid tumors [161]. The tumor penetration of the compound is thus an essential aspect that needs to be studied to obtain optimal drug efficiency [74]. As discussed previously, fluorescence imaging is a complex technique to image spheroids and tumoroids, which has some disadvantages. Additionally, in most circumstances, metal complexes have to be tagged with fluorophores to be detected through this technique, which may affect the distribution of the compound [162,163]. The capacity of the metallodrugs to penetrate tumor models can also be assessed using a combination of analytic and imaging tools, such as laser ablation inductively coupled plasma mass spectrometry (LA-ICP-MS), synchrotron X-ray fluorescence (SXRF), or matrix-assisted laser desorption/ionization mass spectrometric imaging (MALDI-MSI) [69,74,75].

#### 5.1.3. Metabolomics Assays

Omics approaches have been proven to be strong methods for studying drug susceptibility, drug resistance, and mechanisms of action [164]. Metabolic patterns are widely considered accurate predictors of phenotypes and such patterns could be used to predict or better understand the anti-tumoral activity of metallodrugs [165,166]. One approach to studying the metabolites of spheroids after exposure to metallodrugs is using LC-MS-based metabolomics, in which liquid chromatography and mass spectrometry are combined [76,167]. Although not described so far for the study of metallodrugs, other omics studies with different approaches for liquid chromatography and mass spectrometry, seahorse assay, and metabolic imaging have already been used in cancer 3D models [168,169,170,171,172,173].

### 5.2. Selected Examples of the Evaluation of Metallodrugs in 3D Models 

In order to highlight the relevance of the use of 3D models in cancer research, the most relevant applications of these models for metallodrug evaluation will be presented, focusing on discrete metal complexes rather than nanomaterials. From a limited number of reports in the literature, the examples presented next were selected based on the diversity of metals (and metalloids) explored, on the different 3D models and types of assays performed, and on the tumor types (Table 1).

#### 5.2.1. A ruthenium-Based 5-Fluorouracil Complex with Enhanced Cytotoxicity in Colon Carcinoma 

Silva et al. synthesized a novel Ru-based compound, [Ru(5-FU)(PPh_3_)_2_(bipy)]PF_6_, and tested its effect on cell-based models [71]. Complexes containing 5-fluorouracil (5-FU) are commonly used to treat colon and rectal carcinoma, so the cytotoxicity of this compound was studied in human colon carcinoma HCT116 cells [71,174]. 

**Figure 2:**
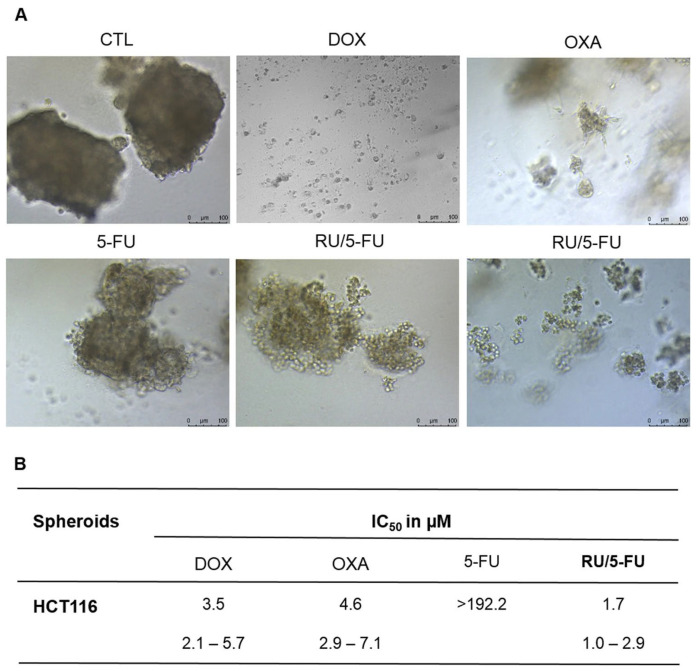
[Ru(5-FU)(PPh_3_)_2_(bipy)]PF_6_ (RU/5-FU) effect on HCT116 spheroids. (**A**) Spheroids observed using light microscopy after incubation with the compounds. (**B**) IC_50_ values in µM and their respective 95% confidence intervals from three separate experiments completed in duplicate, evaluated using the alamar blue test 72 h after incubation with the compounds. The negative control (CTL) was incubated with DMSO. The positive controls comprised doxorubicin (DOX), oxaliplatin (OXA), and 5-fluorouracil (5-FU) [71].

The cytotoxicity of the complex was first tested in several tumoral and healthy cell lines using the alamar blue assay. The cytotoxicity was presented in terms of IC50, which corresponds to the amount of a drug required to inhibit a biological process or response by 50% [175]. The HCT116 cells were determined as the most sensitive to the compound [Ru(5-FU)(PPh_3_)_2_(bipy)]PF_6_, with an IC50 of 1.5 μM. The cytotoxicity was also evaluated in HCT116 spheroids grown at a density of 5000 cells/well in U-bottom low adhesion plates, presenting an IC50 of 1.7 μM, as depicted in Figure 2. As 2D models are reported to be less resistant to drugs than 3D models, it is expected that the IC50 is lower. However, comparing the IC50 obtained for the 2D and 3D models it is possible to see that the difference between the two types of models is very small. So, this complex also presents high cytotoxicity at lower concentrations in 3D models, which suggests that it has great potential for treating solid tumors [71]. This should be further studied in more complex models, such as tumoroids, in order to verify the therapeutic potential of the compound. Overall, [Ru(5-FU)(PPh_3_)_2_(bipy)]PF_6_ presented higher cytotoxicity in tumoral cells than 5-FU in both 2D and 3D cell models [71].

#### 5.2.2. Boron Clusters as a Novel Therapy against Glioblastoma

Metallacarboranes ([M(C_2_B_9_H_11_)_2_]^−^) are 3D aromatic complexes composed of a central metal ion, usually Co or Fe, that is the common vertex of two joined icosahedrons, with interesting advantages in terms of their high stability, water solubility, and redox potential [176,177,178]. Similar to carboranes, these compounds are tunable, but the metal core adds additional features regarding the redox potential and the charge of the molecule [70].

**Figure 3:**
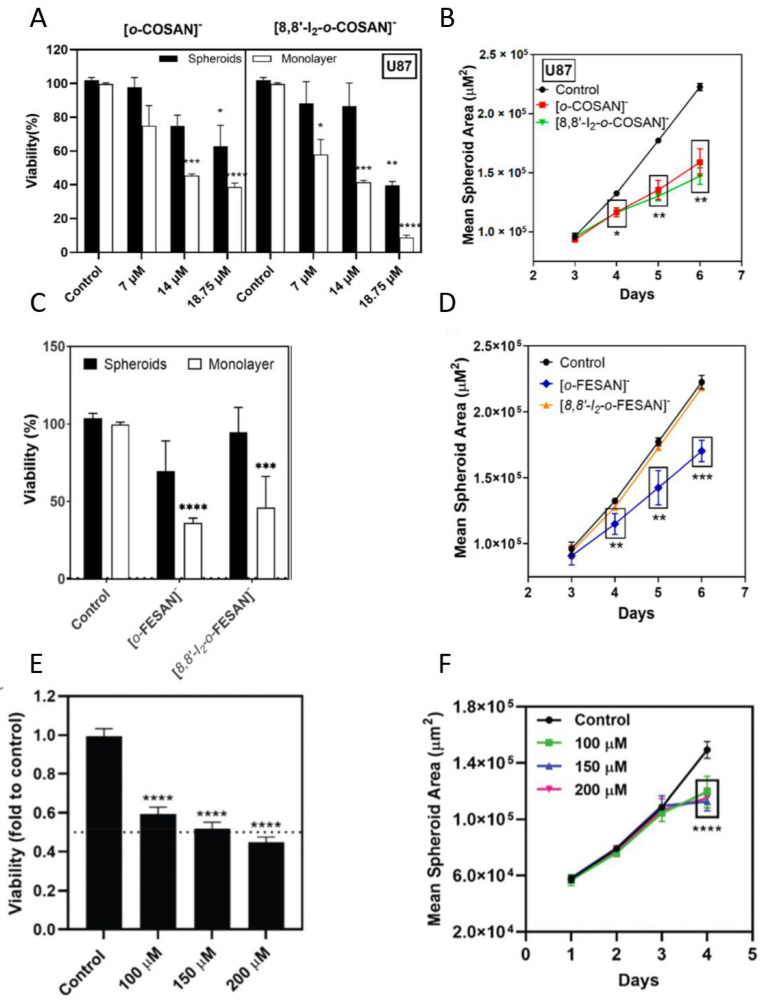
Effects of metallacarboranes in glioblastoma spheroids. (**A**) Viability of spheroids and monolayer-grown cells 72 h after incubation with [*o*-COSAN]^−^ and [8,8′-I_2_-*o*-COSAN]^−^, determined by the APH test. (**B**) Spheroid growth, given by the mean spheroid area as a function of culture time, after incubation with [*o*-COSAN]^−^ and [8,8′-I_2_-*o*-COSAN]^−^. (**C**) Viability of spheroids and monolayer-grown cells 72 h after incubation with [*o*-FESAN]^−^ and [8,8′-I_2_-*o*-FESAN]^−^, determined by the APH test. (**D**) Spheroid growth, given by the mean spheroid area as the function of culture time, after incubation with [*o*-FESAN]^−^ and [8,8′-I_2_-*o*-FESAN]^−^. (**E**) Viability of spheroids 24 h after incubation with Na[*o*-^57^FESAN]^−^, determined by the CellTiter-Glo^®^ 3D Cell Viability Assay. (**F**) Spheroid growth, given by the mean spheroid area in function of culture time, after incubation with Na[*o*-^57^FESAN]. Spheroids or monolayer cultures treated only with the medium were used as the controls. Statistical significance was calculated using one-way ANOVA, followed by Dunnett’s test comparing treated spheroids/cells with control spheroids/cells (* *p* ≤ 0.05, ** *p* ≤ 0.01, *** *p* ≤ 0.001, and **** *p* ≤ 0.0001). Adapted from [70,73,179].

In a glioblastoma cell model, studies have been performed using metallacarboranes with both Fe and Co as a central core, to assess their therapeutic potential [70,73,179]. The cytotoxicity of cobaltabis(dicarbollides) ([*o*-COSAN]^−^ and [8,8′-I_2_-*o*-COSAN]^−^) and ferrabis(dicarbollides) ([*o*-FESAN]^−^ and [8,8′-I_2_-*o*-FESAN]^−^) was first assessed in monolayer cultures of glioblastoma cell lines, and then in spheroids derived from the same cell lines using the APH assay (Figure 3A, and Figure 3C, respectively). The cytotoxicity of another ferrabis(dicarbollide), [*o*-^57^FESAN]^−^, with the potential to treat glioblastoma using the Mössbauer effect, was also evaluated in U87 spheroids (prepared in Nunclon™ Sphera™ ultra-low-attachment 96U-well plates at a density of 1250 cells per well) using the CellTiter-Glo^®^ 3D Viability Assay (Figure 3E) [73]. In general, the results revealed that glioblastoma cells were more sensitive when cultured as a 2D model than when cultured as spheroids, reiterating the relevance of 3D models in cancer research [180]. A physical assessment (area and circularity) of the spheroids was also performed after incubation with the metallacarboranes. The results obtained revealed that all the metallacarboranes tested significantly affected the spheroid growth, resulting in a considerable decrease in the spheroid area, with the exception of Na [8,8′-I_2_-*o*-FESAN] (Figure 3B,D,F). On the other hand, neither compound significantly affected the spheroid circularity. 

Interestingly, the potential radiosensitizing effects of the metallacarboranes mentioned above were also explored, although most of these studies were performed in monolayer cultures. The cobaltabis (dicarbollides) compounds were studied as potential radiosensitizers for boron neutron capture therapy (BNCT), due to their high boron (B) content and high tumor uptake. The concept of BNCT is based on the deposition of high doses of radiation in tumor cells containing 10B-based compounds through a thermal neutron capture reaction that yields high linear energy transfer of α particles able to induce damage in the tumor [179]. 

The radiosensitizing effects of the ferrabis(dicarbollides), [o-FESAN]^−^, and [8,8′-I_2_-o-FESAN]^−^, were further assessed using low dose γ-rays and X-rays, considering that the interaction of Fe with low radiation doses could lead to the emission of photoelectrons, Auger electrons and other secondary electrons, and Compton scattering, increasing the total radiation dose to the tumor cells, consequently causing deleterious effects. Furthermore, the radiosensitizing effect of these compounds was also assessed with proton irradiation considering that the interaction of B with protons in the cells would, theoretically, increase the number of α particles emitted due to a nuclear fusion reaction (proton boron fusion reaction (PBFR)) [70]. Finally, as referred, [*o*-^57^FESAN]^−^ was used to study the radiosensitizing effects of Mössbauer absorption in glioblastoma monolayer cultured cells and spheroids, bearing in mind that the secondary radiation that accompanies the resonance absorption of the Mössbauer radiation, in the ^57^Fe nuclei, is able to produce a powerful local effect on the tumor cells [73].

#### 5.2.3. A New Generation of Anticancer Palladium Agents That Restrain the Growth of Ovarian Cancer Tumoroids 

N-heterocyclic carbenes are found in some of the most active palladium compounds, stabilizing the complex and avoiding its rapid degradation in a biological environment [181,182]. The anticancer activity and cytotoxicity of Pd(II)-η^3^-allyl organometallic complex have been evaluated in two ovarian cancer cell lines, A2780 and SKOV-3, and both the antiproliferative and pro-apoptotic activity appeared to be very promising [183]. Scattolin et al. then studied the effects of these Pd compounds on liver organoids, to evaluate the hepatotoxicity, and ovarian cancer tumoroids, to assess their anticancer activity on more complex models [77]. Regarding the hepatoxicity, the organoids were first characterized by immunohistochemistry using premature and mature hepatocyte markers. Then, it was possible to determine the IC50 of 0.03 ± 0.01 µM for cisplatin and 3 ± 2 µM for the selected Pd compound. This shows that this Pd complex is 100 times less toxic than cisplatin, corroborating with the data from assays on 2D models and supporting the use of the compound in future clinical applications [77]. 

To evaluate the anticancer activity of the selected Pd complex, tumoroids of three patients with high-grade serous ovarian cancer subtypes were developed. Tumor samples of both primary and metastatic sites were used. The morphologic and cytologic similarities between tumoroids and parental tumors were assessed and confirmed using hematoxylin and eosin (H&E) staining. On all of the tumoroids, the Pd compound was active at low concentrations, contrary to what was observed with carboplatin, as shown in Table 2 [77]. The results of these hepatoxicity and anticancer activity assays suggest that this new Pd complex could be interesting for therapeutic applications [77].

#### 5.2.4. Oral Mucosal Organoids as a Potential Platform for Personalized Cancer Therapy

Head and neck squamous cell carcinoma (HNSCC) lines have been cultured in 3D models to overcome the limitations of their 2D counterparts. In this study, Driehuis et al. aimed to study the potential of tumoroids, derived from HNSCC patients, for personalized therapy [78].

Tumoroids were grown from patient-derived HNSCC samples obtained through surgical resections or biopsies and cultured in an appropriate organoid culture medium, and their morphology and histopathology were confirmed using brightfield microscopy, H&E, and immunohistochemistry staining. Following that, 13 tumoroid lines were exposed to cisplatin and carboplatin, two drugs used in the treatment of HNSCC patients. As depicted in Table 3, tumoroids of different origins presented different levels of sensitivity to the compounds [78]. In all of the samples, cisplatin was shown to be more effective at lower doses than carboplatin [78,184]. Moreover, differential gene expression analysis of RNA-seq data was performed to verify if expression profiles could predict how these therapies would work. However, gene enrichment analysis on these gene sets yielded no clear indications of resistance mechanisms.

In clinical trials, the combination of cisplatin with radiotherapy improved relapse-free survival when compared with radiotherapy alone [185]. So here, 10 tumoroid lines were subjected to a variety of radiotherapy treatments in the presence or absence of a toxic dose of cisplatin to determine whether the effects of these treatments were additive or synergistic. When compared with single-agent treatment, the combination of chemotherapy and radiotherapy resulted in increased cell death, with six lines showing increased sensitivity to radiotherapy in the presence of cisplatin. Overall, the radiotherapy response improved in the presence of cisplatin, indicating a synergistic effect [78]. All in all, Driehuis et al. were able to demonstrate that, in vitro, a variable response to cisplatin, carboplatin, and radiotherapy was present, suggesting that these tumoroids have the potential to be used in personalized therapy [78].

#### 5.2.5. Microfocus X-ray Fluorescence Mapping of the Penetration of an Osmium Complex in Ovarian Spheroids 

The organo-osmium complex [(ŋ^6^-p-cym)Os(Azpy-NMe_2_)I]^+^ has shown an anti-tumoral activity both in vitro and in vivo [186,187]. This complex is an inert prodrug that, under intracellular reductive conditions, can be activated by hydrolysis, producing a reactive compound responsible for oxidative stress and apoptosis [188,189]. Sanchez-Cano et al. studied the tumoral cell growth inhibition and drug penetration of the compound on A2780 ovarian carcinoma spheroids. Spheroids were grown in U-bottom cell-repellent 96-well plates at cell densities of 1000–5000 cells/well [75].

**Figure 4:**
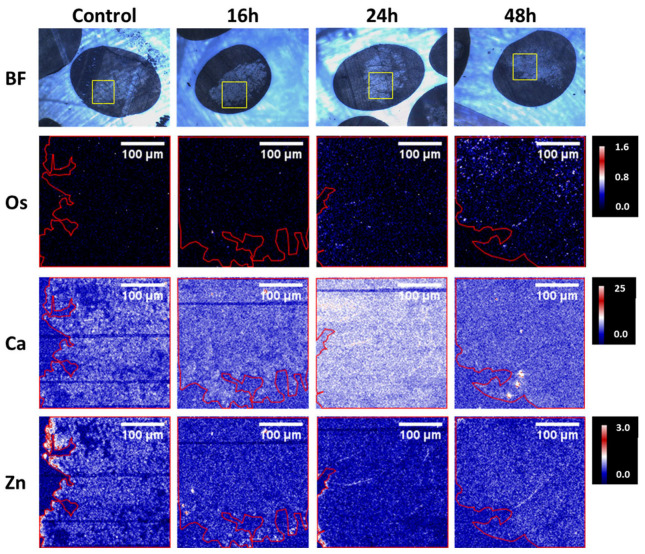
Bright-field images and SXRF elemental maps in A2780 spheroid sections (500 nm) treated for 0, 16, 24, or 48 h with ½ IC50 of [(6-p-cym)Os(Azpy-NMe2)I]^+^. The yellow squares in the bright field images show the spheroid regions analyzed with SXRF. The boundaries of the spheroids are indicated in red in the SXRF elemental maps [75].

Regarding the growth inhibition, it was shown that [(ŋ^6^-p-cym)Os(Azpy-NMe_2_)I]^+^ is more effective than cisplatin, in both 2D and 3D models. Moreover, when the time of exposure increased from 16h to 48h, the differences between the antiproliferative activity of the compound on the 2D and 3D models were minimal, contrary to what happened with cisplatin, in which the biological activity was higher in the monolayer cultures. Such results suggest that the complex was able to penetrate the spheroids in a time-dependent manner [75]. To confirm this hypothesis, a microfocus X-ray fluorescence imaging technique, SXRF, was used to map the distribution of osmium in the spheroids, which is depicted in Figure 4. 

It was possible to observe that [(ŋ^6^-p-cym)Os(Azpy-NMe_2_)I]^+^ reached the tumor core, remaining there for a longer period of up to 48h. The authors suggested a correlation between treatment time and the number of Os atoms at the spheroid cores, possibly due to transport mechanisms [75]. Overall, Sanchez-Cano et al. were able to demonstrate the anti-tumoral potential of [(ŋ^6^-p-cym)Os(Azpy-NMe_2_)I]^+^ against platinum-resistant tumors [75].

#### 5.2.6. Spatially-Resolved Imaging of Platinum Metallodrugs in 3D Spheroids 

**Figure 5:**
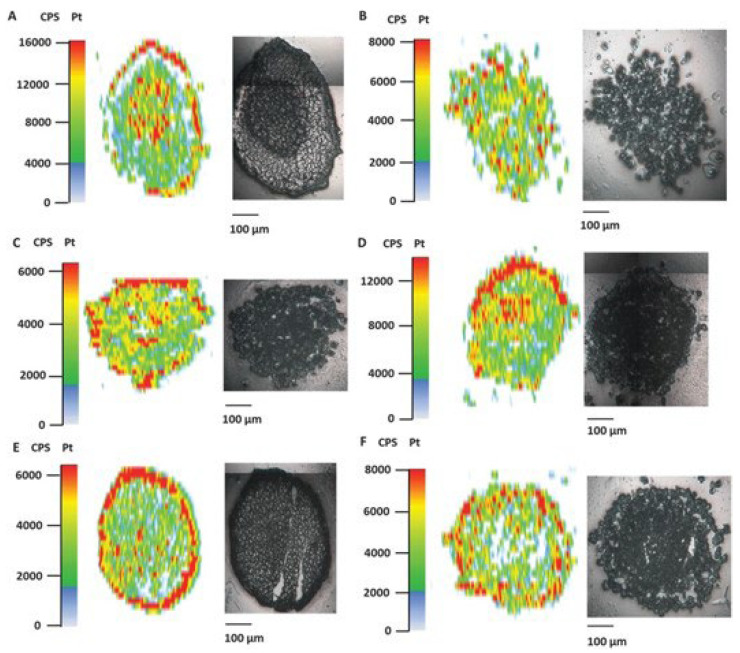
Pt accumulation assessed by LA-ICP-MS in HCT116 and CH1/PA-1 tumor spheroids after treatment (1–5 µM) with Pt (IV) complexes. HCT116 tumor spheroids were incubated with (**A**) satraplatin, (**C**) compound **1**, and (**E**) compound **2**, and CH1/PA1 spheroids with (**B**) satraplatin, (**D**) compound **1**, and (**F**) compound **2.** Reprinted/adapted with permission from Ref. 5562421314339. 2023, Theiner, Sarah; Schreiber-Brynzak, Ekaterina [74].

Driven by the fact that tumor penetration of a metallodrug is a crucial step that has to be studied in order to better understand the tumoral cell’s response, Theiner et al. aimed to apply LA-ICP-MS to study this parameter in two types of tumoral spheroids: colon HCT116 and ovarian CH1/PA-1, exposed to three Pt (IV) compounds. Spheroids derived from HCT116 and CH1/PA-1 were grown in U-bottom 96-well plates at densities of 2000 and 10,000 cells/well, respectively. Using LA-ICP-MS, it was possible to detect the accumulation of the Pt (IV) compounds in specific areas of the spheroids. As can be seen in Figure 5, in the HCT116 spheroids, the Pt enrichment of the three compounds was observed in the periphery and the core, while in CH1/PA-1 spheroids, the accumulation occurred primarily in the periphery (compounds **1** and **2**) and in the central region (compound **1**). The distribution of compound **3** (satraplatin) in the CH1/PA-1 spheroids, did not follow a particular pattern. These findings showed that LA-ICP-MS may be used to analyze the spatial distribution of Pt in heterogeneous structures such as multicellular tumor spheroids [74].

#### 5.2.7. Single-Spheroid Metabolomics

Despite being the subject of several studies, the mechanisms of action of some of the metallodrugs in clinical evaluation are still not fully understood. Studying the metabolome of tumoral cells after incubation with these metallodrugs could be a way of elucidating their anti-tumoral action. Rusz et al. tested spheroids on two different metallodrugs with distinct modes of action on colorectal cancer (HCT116): oxaliplatin and the ruthenium complex KP1339 under clinical trials [76].

**Figure 6:**
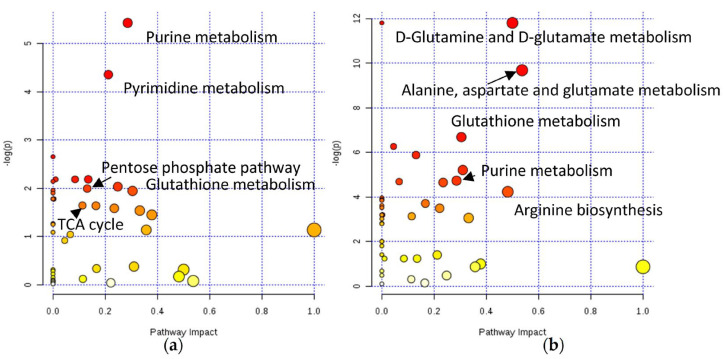
KEGG pathways impacted by (**a**) oxaliplatin and (**b**) KP1339 administration applying module pathway enrichment, topology analysis, and MetaboAnalyst pathway analysis [76].

Spheroids were grown in ultra-low attachment U-bottom 96-well plates at a density of 3000 cells/well. The spheroids metabolome was assessed 24h after exposure to 20 µM and 200 µM of oxaliplatin and KP1339, respectively. In the KP1339-treated samples, 19 metabolites were significantly downregulated compared with only six metabolites in the oxaliplatin-treated samples [76]. These findings were correlated to what was seen in the monolayer culture, where oxaliplatin demonstrated significantly milder effects than KP1339 [190]. The pathway enrichment analysis indicated that oxaliplatin exposure (Figure 6a) altered the purine metabolism and pyrimidine synthesis, being consistent with its established mode of action of DNA targeting, as well as pathways associated with redox stress, such as glutathione metabolism, biosynthesis of coenzyme A, and nicotinamide metabolism [76,191,192]. Furthermore, this work allowed for elucidating the hypothesis that oxaliplatin was also involved in ribosome biogenesis stress [76,192]. On the other hand, KP1339 (Figure 6b) had a distinct mode of action, which resulted in different metabolic perturbations. This drug affected the pathways associated with redox stress, such as glutathione metabolism and purine metabolism, but also unfolded protein response, such as glycerophospholipid metabolism and several amino-acid-metabolism-related pathways [76]. Overall, Rusz et al. were able to develop a protocol for metabolomics studies in tumor spheroid samples, allowing for the comparison of various conditions, such as incubation with different metallodrugs [76].

These examples underline the need for incorporating cancer 3D models in the preclinical testing of metallodrugs, which can support, or not, the results obtained in 2D models. This combined approach can enhance the accurate in vitro assessment of the anti-tumor capabilities of metallodrugs, thereby potentially increasing the success of the process of drug exploration and advancement.

## 6. Conclusions

The majority of clinically tested drugs fail during clinical trials due to inadequate effectiveness or excessive toxicity, resulting in a significant financial loss [193]. As mentioned previously, this occurs in part due to the use of drug testing platforms that are unable to fully represent the tumor and the in vivo microenvironment [194]. 

Different models such as 2D and 3D cellular platforms, and animal models are necessary for the proper preclinical study of metallodrugs for anti-cancer treatments. Nonetheless, most of the metal compounds are still not tested in 3D models. Additionally, as far as we know, the few studies that incorporate 3D models use mostly spheroids. We envisage that in the future this paradigm may change and that 3D models become a regular tool in metallodrug preclinical studies. In addition to the most commonly used spheroids, more advanced platforms, such as organoids and tumoroids, have the potential to make these studies even more relevant by introducing multiple cell types. Complex models such as organ-on-a-chip are already used in drug toxicity assessments for predictive evaluations [156], and tumoroids could even be used in the future as platforms to verify which metallodrug will be more adequate for each patient, covering an important aspect of precision medicine. Tumoroids might also be used to determine whether some subpopulations are more likely than others to respond differently or have side effects to certain metallodrugs depending on their genetic makeup [195]. This is already the case for cystic fibrosis, in which concerted efforts in several European projects have developed and validated intestinal organoids as a clinical tool [196]. All in all, the potential for 3D models as valuable platforms for anti-cancer metallodrug testing has already been proven, and drug development could benefit greatly in the future from the introduction of 3D models as tools in preclinical studies.

## Figures and Tables

**Table 2:** IC50 values for the Pd complex and carboplatin on ovarian cancer tumoroids after 96 h of incubation. Reprinted/adapted with permission from Ref. 5562411110217. 2023, Antonio Togni, Flavio Rizzolio, Nicola Demitri et al. [77].

Compound	IC50 [μM]		
* *	OV-A	OV-B	OV-C
***Carboplatin***	>100	>100	30 ± 8
***Pd complex***	3 ± 2	3 ± 2	2 ± 1

**Table 3:** IC50 values cisplatin and carboplatin in different tumoroid lines, expressed in µmol/L.

Organoid Line	T1	T2	T3	T4	T5	T6	T7	T8	T9	T24	T25	T27	T28
**IC50 cisplatin**	7.9	0.5	6.6	3.0	4.7	7.8	7.0	6.9	12.8	3.8	4.7	6.2	7.7
**IC50** **carboplatin**	19.3	3.0	26.9	8.5	14.9	25.1	55.9	21.7	81.9	13.8	14.9	97.5	55.9

## Data Availability

Not applicable.
